# 

**Published:** 1912-03-09

**Authors:** 


### Appointments.

Dr. W. Knowsley Sibley, M.D. Lond., has been appointed physician to St. John's Hospital for Diseases of the Skin, Leicester Square, W.

The following appointments, to date from April 1, have been made at the Royal Southern Hospital, Liverpool: Mr. T. H. Martin, M.B., Ch.B. Liverpool, and Mr. Robert Findlay, M.B., Ch.B. Glasgow, to be house physicians; Mr. Naughton Dunn, M.B., Ch.B. Aberd., Mr. Quintus Madge, B.A. Camb., M.R.C.S., L.R.C.P., and Mr. Hilton Parry, M.B., Ch.B. Liverpool, to be house surgeons.

Dr. Leonard Gregory Parsons, M.D., B.S. Lond., M.R.C.P. Lond., has been appointed assistant physician for a term of three years at the General Hospital, Birmingham. Mr. W. A. L. Holland and Mr. Ernest Wilson Hirst were appointed surgical casualty officers. Dr. Parsons is hon. pathologist to the Queen's Hospital, assistant lecturer on pathology at the University, and physician to out-patients at the Children's Hospital.

Mr. Harold H. Tanner, M.B., B.S. Lond., has been appointed house physician at the Hampstead General and North-West London Hospital, and Mr. Martin W. Littlewood, M.R.C.S., L.R.C.P. Eng., B.S. Lond., house surgeon.

### Buildings Contemplated and in Progress.

Brighton.—It is proposed to complete the Throat and Ear Hospital as originally intended by the erection of an east wing at a cost of £2,000.

Bridlington.—On the advice of the medical officer the Town Council are considering the advisability of erecting a sanatorium.

Cardiff.—The Cardiff Guardians have acquired land upon which to erect a new infirmary and possibly also a phthisical block.

Croydon.—The Local Government Board have sanctioned the borrowing of £2,500 for the provision of a boiler house and heating apparatus at the Borough Hospital.

Carnarvon.—Three close tenders have been received for the erection of a new infirmary for the Guardians. The lowest is £6,848, the highest £6,896. The architect's estimate was £6,932.

Exeter.—The City Council are endeavouring to raise a loan of £15,500 for the extension of the Infectious Diseases Hospital.

Elham.—Forms of tender may be obtained from the architect, Mr. A. R. Bowles, of Folkestone, for the erection of a new female block and nurses' home at the Guardians' Workhouse, near Lyminge.

Edinburgh.—The Dean of Guild Court have approved plans for conversion of premises in Chalmers Street into a nursing home; £9,000 is the estimated cost of the works, which are being carried out under the supervision of Mr. T. D. Rhind, of Edinburgh.

Sligo.—The Guardians are about to have gas installed at the workhouse for lighting purposes.

### Florence Nightingale Memorial.

The following contributions have been received by Mr. G. Q. Roberts during the past week:—

Amy Lady Tate, £25; Miss Clara Thomas (per Sir Henry Burdett), £20 (£10 Statue, £10 Annuities); Army Service Corps, Aldershot (in addition to £5 already received), £7 5s. 7d.; collection in Girls' School Chapel, Christ's Hospital, Hertford, £5 5s.; Sir John Ramsden, Bart., £5; 2nd Batt. Alexandra P.W.O. Yorks Regt., £5; Miss Philip Smith, £3 3s.; Lady Holland, £2 2s.; Lady Smyth, £2 2s.; Hon. Seth Low, Mayor of New York, £2 2s.; Lieut.-Col. F. J. Heyworth, £2 2s.; collection at annual meeting of Worcester Branch, N.U.W.S.S. (Annuities), £1 10s. 6d.; Sir J. N. Barran, Bart., £1 1s.; Miss Rosalind Paget, £1 1s.; Mrs. Vaughan (Annuities), £1 1s.; Francis N. Smith, Esq., £1 1s.; Lieut.-Gen. Willoughby, 10s. 6d.; R. C. and L. C., 3s. 6d.; Mrs. Beauchamp, 3s. 6d.

